# Household environment and symptoms of childhood acute respiratory tract infections in Nigeria, 2003–2013: a decade of progress and stagnation

**DOI:** 10.1186/s12879-018-3207-5

**Published:** 2018-07-03

**Authors:** Joshua Odunayo Akinyemi, Oyewale Mayowa Morakinyo

**Affiliations:** 1 Department of Epidemiology and Medical Statistics, Faculty of Public Health, College of Medicine, University of Ibadan, Ibadan, Nigeria; 2 Demography and Population Studies Programme, Schools of Public Health and Social Sciences, University of the Witwatersrand, Johannesburg, South Africa; 3 Department of Environmental Health Sciences, Faculty of Public Health, College of Medicine, University of Ibadan, Ibadan, Nigeria

**Keywords:** Acute respiratory tract infection symptoms, Household environment, Under-five children, Nigeria

## Abstract

**Background:**

Acute respiratory infections (ARIs) remains a disease of public health importance in Nigeria. Though, previous studies have identified factors associated with childhood ARI symptoms, the progress made in reducing the burden of this major childhood morbidity in the past decade in Nigeria has not been quantified. Therefore, this study describes the trends in the prevalence and factors associated with ARI symptoms among under-five (U5) children in Nigeria between years 2003 and 2013.

**Methods:**

A retrospective cross-sectional analysis of nationally representative data from the Nigeria Demographic and Health Surveys (NDHS) for years 2003, 2008 and 2013 was done. The study sample included women of reproductive age who had U5 children presenting with a cough accompanied with short rapid breaths in the last 2 weeks prior data collection. Data were analysed using complementary log regression model.

**Results:**

Prevalence of ARI symptoms were 10.3, 4.6 and 3.8% for years 2003, 2008 and 2013 respectively. The use of unclean cooking fuel was not associated with ARI symptom in 2003 and 2008, but in 2013 (OR = 2.50, CI: 1.16–5.42). Living in houses built with poor quality materials was associated with higher risk of ARI symptoms in 2008 (OR = 1.34, CI: 1.11–1.61) and 2013 (OR = 1.59, CI: 1.32–1.93). Higher risk of ARI symptoms was also associated with younger child’s age, Northern regions and household wealth quintile between 2003 and 2013.

**Conclusions:**

Though there has been a significant progress in the reduction of the prevalence of ARI symptoms between 2003 and 2013, the same could not be said of household environmental risk factors. Interventions to reduce the contributory effects of these factors to the occurrence of ARI symptoms should be instituted by government and other relevant stakeholders.

## Background

Acute respiratory infection (ARI), a disease of public health significance, is a collection of diseases caused by a heterogeneous mix of organisms that affects the human airways [1]. ARI is the third largest singular cause of mortality in both developed and developing countries [2, 3] resulting in about 1.6 million deaths among children under five annually [4, 5]. Children living in developing countries are ten to fifty times more likely to die from ARI than those living in developed countries [6].

In developing countries, about 70% of ARI morbidities are linked to ARI occurrence in under-five children [7], with a child experiencing an average of 5–6 episodes per year [8]. A World Health Organisation (WHO) study in developing countries reported an ARI incidence rate of 15–21% in children under-five [9], with greater than two third of the ARI global prevalence in Africa [10]. The incidence of ARI in children is one of the major reasons for paediatric outpatients’ visits and hospitalisation [11] in developing countries.

Pneumonia and bronchiolitis are the commonest ARIs in children [12]. It’s the foremost cause of childhood deaths, annually responsible for about 2 million deaths among children under 5 years of age [13], translating to one death every 23 s [4]. In the year 2015, pneumonia was responsible for 15% of all under-five deaths and 920,000 childhood deaths [10]. In Nigeria, the WHO estimates that 868,000 under-five children died from pneumonia in 2010, killing more than 140,000 annually [4]. This number is the highest in Africa [4], and the second highest estimated number of childhood deaths from ARI in the world [14].

In Nigeria, the identified risk factors for ARIs in children include age and gender of the child, nutritional status, wealth index, parental educational status, large family size, exposure to biomass fuel, and parental smoking status. Other factors include daycare attendance, insufficient breastfeeding practice, poor immunization status, attendance to day care centers, and overcrowding [15, 16]. Moreover, different viral types have been associated with ARI among Nigerian children. Akinloye et al. reported that viruses such as human rhinoviruses, and parainfluenza viruses, with influenza virus C, human metapneumovirus, human bocavirus and Adenoviruses, were detected in 77% of the 246 Nigerian children presenting with ARI [17]. Elsewhere in Nigeria, respiratory syncytial virus B, Parainfluenza virus 4, and enterovirus where identified in 93.8% (15/16) of clinical samples [15].

Though, previous studies have identified some of the factors associated with childhood ARI symptoms in Nigeria, the progress made in the efforts to reduce the threat of this major childhood morbidity in the past 10 years has not be quantified. This study is aimed at addressing this gap. Therefore, the main objective of this study is to describe the trends in the prevalence and factors associated with ARI symptoms among under-five children in Nigeria between 2003 and 2013.

## Methods

### Study setting

Nigeria, a federal republic in West Africa, is administratively divided into 36 states and a federal capital territory (FCT). These states are further divided into six geopolitical zones: North-West, North-Central, North-East, South-East, South-West, and South-South. In each of the geopolitical zones, the people are homogeneous and unique in their ways of life.

### Data source

This study is a retrospective cross analysis of nationally representative data from the Nigeria Demographic and Health Surveys (NDHS) for 2003, 2008 and 2013. In the NDHS, women of reproductive age (15–49 years) are interviewed about their reproductive health, children health and other related topics. Selection of survey respondents involved a stratified two-stage cluster design in which all the census enumeration areas (EAs) in the country are stratified into rural and urban areas. A specified number of EAs was then selected from each stratum. Households in selected EAs were listed and 45 were systematically selected in each one [18]. Data were collected from eligible women at selected households with the aid of a pretested questionnaire by trained interviewers. Data collected from women about their under-five children were specially processed to constitute the kids recode file which was utilised for analysis in this study. The kids recode file contain household, maternal and child-related variables. Detailed description of field procedures are well stated in the full report of the surveys [18–20].

### Derivation of variables

Three sets of variables were analysed: household environment, maternal and children characteristics. The variables were created from the household and children recode file respectively for each of the three surveys. Variables for the household environment include the type of cooking fuel used in the household; smoking by adults in the household; source of drinking water; type of toilet, quality of housing material and over-crowding in the sleeping room. Cooking fuel was categorised as clean (electricity, liquefied petroleum gas, natural gas or biogas) and unclean (coal, ignite, charcoal, wood, kerosene, animal dung, straw, shrubs, and grass). Source of drinking water was classified as improved (piped into dwelling/yard/plot, public tap/standpipe, tube-well or borehole, protected well and spring, rain water, and bottle water) or unimproved (unprotected well and spring, tanker truck/cart with drum, surface water, sachet water, and other sources). Similarly, toilet type was either improved (flush/pour flush to piped sewer system, septic tank or pit latrine, ventilated improved pit latrine, pit latrine with slab, composting toilet) or unimproved (flush/pour flush not to sewer/septic tank/pit latrine, pit latrine without slab/open pit, bucket, hanging toilet/latrine, no facility/bush/field) [18, 21, 22].

The quality of housing material was derived from a composite index based on the type of floor, wall and roof materials. Floor material was coded 1 if made of cement, carpet/rug, ceramic tiles, vinyl asphalt strips, parquet or polished wood, and 0 otherwise. Also, wall material was coded 1 if made of cement, stone with lime/cement, cement blocks or bricks and 0 if otherwise. For roof material, the following were coded as improved (1): cement, roofing shingles, calamine/cement fibre, ceramic tiles, metal/zinc. To derive the final categories for quality of housing material, the scores for the floor, wall, and roof were aggregated and re-coded as follows: good (3), average (2) and poor (0/1). Sleeping room was deemed over-crowded if the average number of person per sleeping room in the household was greater than or equal to three. The groupings of environmental factors were adapted from the year 2013 Nigeria National Demographic Health Survey (NDHS) and the 2010 WHO and UNICEF document on progress on sanitation and drinking water [18, 21]. For the 2003 survey, there was no data collected on the type of roofing material. As a result of which the variable for housing quality was not derived.

The maternal characteristics which were extracted from the children recode file were: maternal age, education, occupation, household wealth quintile, type of residence, and geopolitical region. Children characteristics included current age in months, sex, birth order and stunting which was an indicator of chronic malnutrition. The outcome variable was ARI symptom which was derived from the response to two questions on whether under-five had a cough in the last 2 weeks and if a cough was accompanied by short rapid breaths.

### Analysis

Data for each survey (2003, 2008 and 2013) were analysed separately. The analysis involved the use of frequencies and percentages to describe all the study variables. Next, univariate models were fitted to describe the relationship between the outcome and explanatory variables. At the third step in the analysis, two models were fitted. In the first model, variables for the household environment were included in the model (Model I). Secondly, other maternal and children characteristics were added to the first model. As such the second which is a full model was used to identify the independent factors associated with childhood ARI symptoms. The complementary log regression model with robust standard errors was employed because of the outcome variables. This model is suitable for situations in which the dependent variable is dichotomous and its proportion in the analytical sample is below 10% [23]. Model coefficients were exponentiated to derive odds ratio with their 95% confidence interval. Stata SE Version 12.0 was used for all analyses. Sample weights were computed and applied to account for the complex sample design of the surveys.

The model is of the form:

$$ \Pr \left({y}_j=1|{x}_j\right)=1-\exp \left[-\exp \left({x}_j\beta \right)\right] $$

Where *y*_*j*_ = presence of ARI symptom in child *j.*

*x*_*j*_ = vector of covariates (explanatory variables).

β = coefficients for the covariates (*x*_*j*_).

## Results

### Household characteristics

The household characteristics of all under-five children in the study sample and those who had symptoms of ARI are presented in Table 1. For the overall sample, most under-five children lived in households using unclean fuel for cooking with percentages ranging from 97.5% in 2003 to 98.4% in 2013 (Table 1). The proportions of households in which adult members smoke declined over the years (2003–31.6%, 2013–6.9%). The quality of housing material remained almost the same between 2008 and 2013 with about 40% of children dwelling in buildings with good quality housing materials. Similarly, slightly more than half of the under-fives sleeps in the over-crowded room and the proportion also remained the same over the study period. A little above 40% of children live in poor households while two-third of the entire sample reside in rural areas. Under-five children from the Northwest and Northeast regions constituted one-third and about one-fifth of the total sample across the three surveys. The prevailing season at the time of data collection was inferred from the month of the survey. In 2003 and 2008, 70.8 and 99.8% of data were collected during the rainy season (May to October) while in 2013, it was 25.2%.

**Table 1:** Percentage of under-five children according to ARI symptoms status, household environmental and other background characteristics, Nigeria, 2003–2013

Variables	2003 (*n* = 5445)		2008 (*n* = 24,975)		2013 (*n* = 28,950)	
ARI symptoms	All children	Children with ARI symptoms	All children	Children with ARI symptoms	All children	Children with ARI symptoms
ARI symptoms	550 (10.3)		1159 (4.6)		1095 (3.8)	
Household environment						
Unclean cooking fuel	97.5	99.0	98.1	98.8	98.4	99.1
Cooking inside the house	NA					
Smoking by household members	31.6	32.6	0.2	0.4	6.9	10.1
Improved source of drinking water	18.2	11.9	51.0	46.8	56.6	52.0
Improved toilet	75.9	76.5	51.7	44.3	51.2	44.8
Quality of housing material						
Poor	NA		42.9	48.9	41.2	50.0
Average	NA		14.3	15.1	16.4	17.1
Good	NA		42.8	36.0	42.4	32.9
Over-crowded sleeping room	58.1	55.6	58.5	54.6	56.2	53.3
Wealth quintile						
Poor	42.6	48.4	44.8	51.7	45.3	51.3
Middle	20.0	23.5	19.2	19.6	19.1	24.2
Rich	37.3	28.1	36.0	28.7	35.6	24.5
Residence						
Rural	69.7	77.1	69.2	72.0	64.1	70.0
Urban	30.3	22.9	30.8	28.0	35.9	30.0
Region						
North West	34.0	29.1	30.4	19.4	36.2	16.7
North East	22.9	36.1	16.0	34.3	17.4	42.2
North Central	14.6	9.6	13.8	10.5	13.9	14.5
South East	6.5	4.0	9.7	11.0	8.9	12.7
South South	12.8	15.0	13.3	18.7	9.5	9.9
South West	9.2	6.0	16.9	6.2	14.1	4.0
Season of interview						
Dry	29.2	39.2	0.8	0.2	62.8	74.8
Rainy	70.8	60.8	99.8	99.8	37.2	25.2

Except for a few variables, the household characteristics of children with ARI symptoms was like that of the overall sample. The variables for which the distributions differ were quality of housing material, wealth quintile, type of place of residence, and geopolitical region. Close to half (50.0%) of children with ARI symptoms lived in buildings with poor quality housing materials in 2008 and 2013. Similarly, about half of under-five children in whom ARI symptoms were reported to belong to households in the poor wealth quintile while a quarter was from rich households. Slightly more than 70% of children with ARI symptoms were from rural areas and this was consistent over time. Of all the six geopolitical regions, the Northeast (2003–36.1%, 2008–34.3%, 2013–42.2%) has the highest proportion of ARI symptoms among under-fives. The seasonal pattern suggests that the percentage of children affected during the dry season was more than the overall percentage surveyed in the same season.

### Maternal and children characteristics

Majority of the mothers were aged 20–39 years and the distribution was the same in the three surveys. About half of the children had mothers with no formal education. The percentage of under-five whose mothers were involved in professional/services occupation increased slightly from 5.0% in 2003 to 8.5% in 2013 (Table 2). A larger proportion of the children were aged at least 36 months. Distribution of the birth order revealed that about one-third were second or third births. Chronic malnutrition was prevalent among 31.5% in 2003 and 33.1% in 2013 (Table 2). Distribution of maternal age among children with ARI symptoms was also similar to that of the overall sample with about half having mothers aged 20–29 years, ditto for maternal education and occupation (Table 2). Age pattern among children with ARI symptoms showed that they were predominantly below 23 months.

**Table 2:** Percentage of under-five children according to ARI symptoms status, maternal and child characteristics, Nigeria, 2003–2013

Variables	2003 (*n* = 5445)		2008 (*n* = 24,975)		2013 (*n* = 28,950)	
	All children	Children with ARI symptoms	All children	Children with ARI symptoms	All children	Children with ARI symptoms
ARI symptoms	550 (10.3)		1159 (4.6)		1095 (3.8)	
Maternal characteristics						
Age of mother						
< 20	6.9	9.9	5.2	6.8	4.9	5.6
20–29	51.4	54.5	48.1	53.2	47.8	50.8
30–39	32.9	28.0	36.6	31.1	37.5	32.1
> = 40	8.7	7.6	10.1	8.9	9.8	11.5
Education						
None	50.0	53.6	45.4	47.8	48.2	44.5
Primary	23.6	26.0	23.3	23.8	19.2	23.5
Secondary/higher	26.4	20.4	31.3	28.5	32.6	31.9
Occupation						
Not working	34.8	33.4	30.7	29.6	29.6	30.9
Professional/services	5.0	3.8	7.5	5.4	8.5	8.1
Sales	38.9	43.1	33.5	28.2	39.2	30.2
Agriculture/manual	21.2	19.7	28.2	36.8	22.7	30.8
Child characteristics						
Age (months)						
0–11	24.9	35.0	22.9	24.2	21.6	24.7
12–23	18.7	21.4	19.8	28.1	20.4	29.3
24–35	19.6	18.8	18.6	19.5	19	21.1
> = 36	36.8	24.8	38.7	28.2	39.1	24.9
Sex						
Male	50.8	51.7	50.5	51.2	50.1	50.5
Female	49.2	48.3	49.5	48.8	49.9	49.5
Birth order						
1	20.3	22.4	19.3	21.0	19.4	17.1
2–3	31.7	30.5	33.7	32.4	32.8	31.4
4–5	22.3	24.4	23.7	21.2	23.7	22.3
6+	25.8	22.7	23.4	25.4	24.1	29.1
Chronic malnutrition						
Yes	31.5	28.8	30.6	34.9	33.1	33.6
No	68.5	71.2	69.4	65.1	66.9	66.4

### Bivariate association between household, maternal, and children characteristics and ARI symptoms

**Fig. 1:**
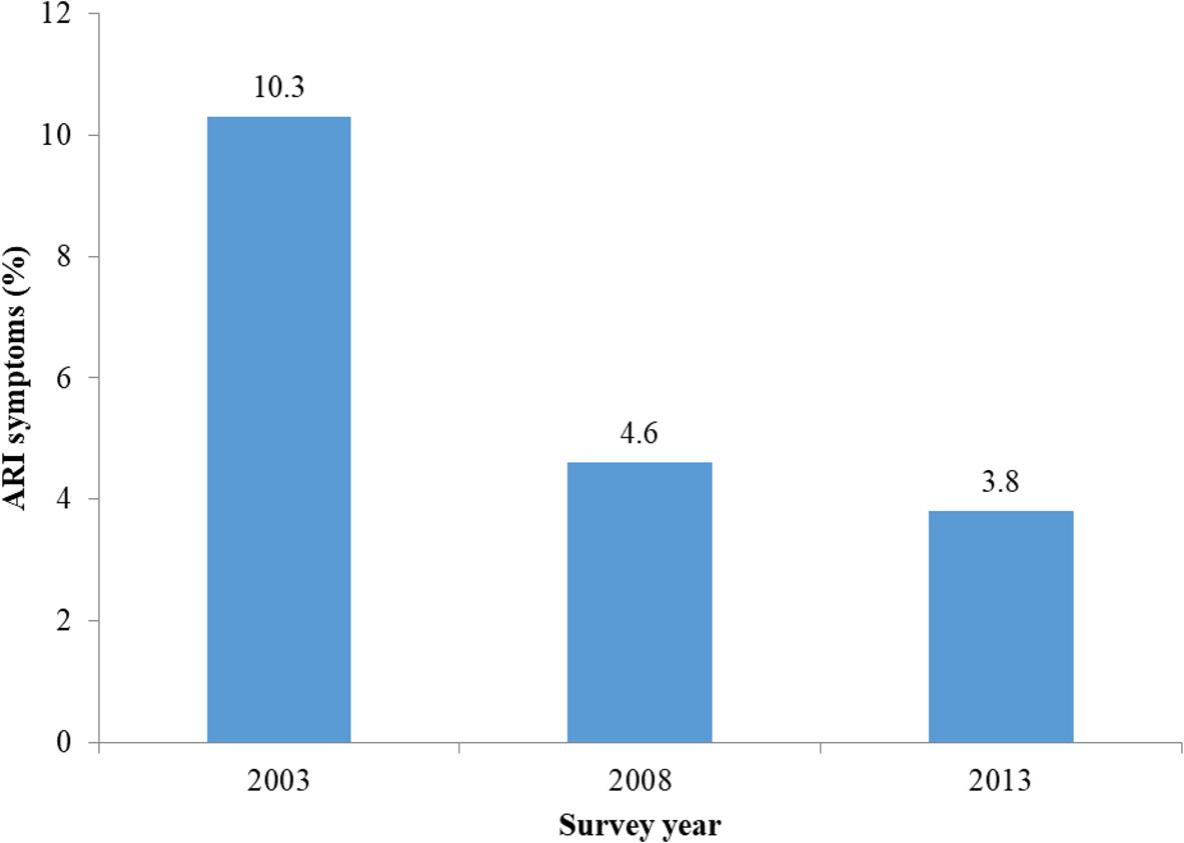
Prevalence of ARI symptoms, 2003–2013. It shows a reduction in the prevalence of ARI symptoms among Nigeria under-five children between year 2003 and 2013

Figure 1 shows that the prevalence of ARI symptoms were: 2003 (10.3%; CI: 9.5–11.1), 2008 (4.6%; CI: 4.4–4.9) and 2013 (3.8%; CI: 9.5–11.1). Table 3 showed the unadjusted odds ratio for the relationship between childhood ARI symptoms and other household and background characteristics. While the use of unclean cooking fuel was not associated with ARI symptom in 2003 and 2008, the reverse was the case in 2013 (OR = 2.50, CI: 1.16–5.42). The odds of ARI symptom were found to be higher among children who lived in households with adult members who smoke cigarette/tobacco or other substances. Availability of improved source of drinking water and toilet was associated with lower odds or ARI symptoms throughout the study period.

**Table 3:** Unadjusted Odds Ratio for the relationship between ARI symptoms, household environmental characteristics, and maternal and child characteristics, Nigeria, 2003-2013

Variables	2003 (*n* = 5445)	2008 (*n* = 24,975)	2013 (*n* = 28,950)
	OR (95% CI)	OR (95% CI)	OR (95% CI)
Household environment			
Unclean cooking fuel	0.99 (0.98–1.00)	1.00 (0.99–1.01)	2.50 (1.16–5.42)*
Cooking inside the house	NA		
Smoking by household members	1.08 (0.88–1.33)	2.24 (0.94–5.32)	1.20 (0.89–1.61)
Improved source of drinking water	0.66 (0.49–0.89)*	0.86 (0.73–1.03)	0.79 (0.67–0.96)*
Improved toilet	0.89 (0.70–1.14)	0.83 (0.71–0.96)*	0.79 (0.67–0.95)*
Quality of housing material			
Poor	NA	1.34 (1.11–1.61)*	1.59 (1.32–1.93)*
Average	NA	1.28 (1.03–1.59)*	1.48 (1.19–1.85)*
Good	NA	1.00	1.00
Over-crowded sleeping room	0.95 (0.79–1.14)	0.87 (0.77–1.00)	0.93 (0.79–1.10)
Wealth quintile			
Poor	1.45 (1.13–1.85)*	1.40 (1.15–1.70)*	1.78 (1.43–2.22)*
Middle	1.42 (1.09–1.84)*	1.30 (1.06–1.61)*	1.88 (1.49–2.36)*
Rich	1.00	1.00	1.00
Residence			
Rural	1.00	1.00	1.00
Urban	0.82 (0.63–1.06)	0.93 (0.75–1.16)	0.74 (0.58–0.93)*
Region			
North West	1.29 (0.83–2.02)	1.67 (1.13–2.48)*	4.55 (2.69–7.67)*
North East	2.38 (1.56–3.62)*	5.28 (3.60–7.75)*	13.00 (8.23–20.53)*
North Central	0.98 (0.59–1.65)	1.87 (1.24–2.81)*	1.89 (1.13–3.15)*
South East	1.34 (0.78–2.31)	3.20 (2.13–4.83)*	7.00 (4.29–11.42)*
South South	1.71 (1.05–2.79)*	3.26 (2.18–4.88)*	4.55 (2.79–7.42)*
South West	1.00	1.00	1.00
Season of interview			
Dry season	1.68 (1.32–2.14)*	1.15 (0.96–1.38)	1.58 (1.21–2.06)*
Rainy season	1.00	1.00	1.00
Maternal characteristics			
Age of mother			
< 20	1.51 (0.94–2.43)	1.41 (0.99–2.01)	1.24 (0.89–1.70)
20–29	1.09 (0.76–1.56)	1.23 (0.96–1.57)	1.03 (0.82–1.29)
30–39	1.06 (0.74–1.52)	1.01 (0.79–1.30)	0.86 (0.69–1.06)
> = 40	1.00	1.00	1.00
Education			
None	1.43 (1.11–1.85)*	1.21 (0.99–1.47)	1.01 (0.83–1.22)
Primary	1.34 (1.03–1.780*	1.22 (1.01–1.47)*	1.24 (1.02–1.50)*
Secondary/higher	1.00	1.00	1.00
Occupation			
Not working	0.99 (0.57–1.69)	1.24 (0.89–1.74)	1.31 (0.98–1.74)
Professional/services	1.00	1.00	1.00
Sales	1.05 (0.63–1.77)	1.17 (0.84–1.62)	0.85 (0.65–1.11)
Agriculture/manual	1.01 (0.59–1.69)	1.73 (1.22–2.43)*	1.50 (1.14–1.99)*
Child characteristics			
Age (months)			
0–11	1.99 (1.58–2.51)*	1.43 (1.22–1.67)*	1.83 (1.57–2.14)*
12–23	1.80 (1.40–2.32)*	1.92 (1.67–2.20)*	2.36 (2.02–2.76)*
24–35	1.46 (1.12–1.89)*	1.35 (1.14–1.60)*	1.78 (1.49–2.12)*
> = 36	1.00	1.00	1.00
Sex			
Male	1.03 (0.86–1.22)	0.99 (0.88–1.11)	1.04 (0.93–1.17)
Female	1.00	1.00	1.00
Birth order			
1	1.00	1.00	1.00
2–3	0.96 (0.76–1.22)	0.86 (0.74–1.00)	0.99 (0.83–1.17)
4–5	1.09 (0.85–1.39)	0.83 (0.69–1.01)	1.01 (0.84–1.22)
6+	1.03 (0.79–1.33)	1.02 (0.85–1.23)	1.29 (1.06–1.57)*
Chronic malnutrition			
Yes	0.93 (0.78–1.12)	1.19 (1.06–1.36)*	1.03 (0.88–1.20)
No	1.00	1.00	1.00

*p <0.05 (statistically significant)

The results further revealed that poor quality of housing materials was associated with higher risk of ARI symptoms among under-five children in 2008 (OR = 1.34, CI: 1.11–1.61) and 2013 (OR = 1.59, CI: 1.32–1.93). Maternal characteristics associated with higher risk of ARI symptoms in children include agriculture/manual occupation and poor household wealth quintile. These associations remained consistent especially in the recent two surveys (i.e. 2008 and 2013). Regional difference in the odds of ARI symptoms widened over time especially in the North West and North East versus South West.

### Multivariate analysis

Table 4 presents the adjusted odds ratio for the factors associated with ARI symptoms. Results for the household characteristics showed that their pattern of association with childhood ARI symptoms remained virtually the same over the years 2003–2013. This was evident from the overlapping confidence intervals for the odds ratios. Results for 2013 suggest that household use of unclean cooking fuel is associated with greater odds of ARI symptoms (aOR = 1.44, CI: 0.66–3.13) though not statistically significant. The presence of a smoker in the households increased the likelihood of ARI symptom while the availability of an improved source of drinking water and toilet reduced it. Model I (household environment) for 2008 and 2013 showed that poor quality of housing material was significantly associated with higher odds of ARI symptoms among under-five children. However, controlling for maternal and children characteristics partly explained the relationship such that statistical significance disappeared though the odds ratio was still greater than 1.00.

**Table 4:** Adjusted Odds Ratio for the relationship between ARI symptoms, household environmental characteristics, and maternal and child characteristics

Variables	2003		2008		2013	
	OR (95% CI)	OR (95% CI)	OR (95% CI)	OR (95% CI)	OR (95% CI)	OR (95% CI)
	Model 1	Model 2	Model 1	Model 2	Model 1	Model 2
Household environment						
Unclean cooking fuel	0.99 (0.98–1.00)	0.99 (0.98–1.00)	1.00 (0.99–1.01)	1.00 (0.99–1.01)	1.98 (0.92–4.28)	1.44 (0.66–3.13)
Cooking inside the house	NA					
Smoking by household members	1.09 (0.89–1.34)	1.11 (0.91–1.35)	2.27 (0.94–5.48)	2.72 (1.07–6.88)*	1.18 (0.89–1.57)	1.13 (0.85–1.51)
Improved source of drinking water	0.67 (0.49–0.91)*	0.79 (0.58--1.09)	0.96 (0.81–1.14)	1.14 (0.96–1.35)	0.92 (0.76–1.11)	1.05 (0.88–1.25)
Improved toilet	0.94 (0.74–1.19)	0.86 (0.64–1.17)	0.89 (0.77–1.04)	1.01 (0.85–1.19)	0.93 (0.77–1.12)	0.96 (0.80–1.16)
Quality of housing material						
Poor	NA	NA	1.27 (1.05–1.54)*	1.00 (0.73–1.37)	1.48 (1.22–1.80)*	1.35 (0.98–1.86)
Average	NA	NA	1.25 (0.99–1.56)	1.11 (0.87–1.42)	1.42 (1.13–1.78)*	1.19 (0.93–1.52)
Good	NA	NA	1.00	1.00	1.00	1.00
Over-crowded sleeping room	0.96 (0.81–1.15)	0.95 (0.79–1.13)	0.88 (0.77–1.00)	0.92 (0.81–1.06)	0.92 (0.78–1.09)	1.05 (0.89–1.24)
Wealth quintile						
Poor		1.07 (0.76–1.50)		1.16 (0.79–1.70)		1.21 (0.84–1.73)
Middle		1.19 (0.89–1.57)		1.12 (0.87–1.45)		1.48 (1.14–1.91)*
Rich		1.00		1.00		1.00
Residence						
Rural		1.00		1.00		1.00
Urban		0.96 (0.72–1.29)		1.09 (0.87–1.39)		0.99 (0.79–1.26)
Region						
North West		1.24 (0.77–2.01)		1.61 (1.04–2.48)*		3.89 (2.31–6.55)*
North East		2.26 (1.43–3.58)*		4.89 (3.20–7.47)*		11.53 (7.05–18.86)*
North Central		0.89 (0.54–1.51)		1.69 (1.11–2.59)*		1.69 (0.96–2.99)
South East		1.32 (0.77–2.28)		3.15 (2.07–4.780*		5.88 (3.56–9.72)*
South South		1.71 (1.05–2.77)*		3.21 (2.13–4.83)*		3.86 (2.33–6.39)*
South West		1.00		1.00		1.00
Season of interview						
Dry season		1.75 (1.39–2.21)*		–		1.54 (1.25–1.91)*
Rainy season		1.00				1.00
Maternal characteristics						
Age of mother						
< 20		1.07 (0.58–1.98)		1.22 (0.80–1.86)		1.19 (0.79–1.81)
20–29		0.99 (0.64–1.53)		1.29 (0.98–1.72)		1.11 (0.85–1.45)
30–39		1.01 (0.71–1.45)		1.06 (0.83–1.36)		0.89 (0.72–1.12)
> = 40		1.00		1.00		1.00
Education						
None		1.29 (0.91–1.83)*		1.03 (0.82–1.28)		0.63 (0.52–0.78)*
Primary		1.32 (0.97–1.80)		1.09 (0.90–1.34)		0.92 (0.76–1.12)
Secondary/higher		1.00		1.00		1.00
Occupation						
Not working		0.68 (0.38–1.24)		0.93 (0.66–1.33)		0.85 (0.63–1.15)
Professional/services		1.00		1.00		1.00
Sales		0.82 (0.47–1.44)		1.04 (0.74–1.47)		0.80 (0.61–1.06)
Agriculture/manual		0.77 (0.44–1.35)		1.32 (0.92–1.87)		0.99 (0.74–1.33)
Child characteristics						
Age (months)						
0–11		2.04 (1.58–2.63)*		1.43 (1.22–1.69)*		1.80 (1.52–2.12)*
12–23		1.86 (1.43–2.41)*		1.88 (1.64–2.18)*		2.32 (1.97–2.72)*
24–35		1.51 (1.15–1.98)*		1.32 (1.12–1.57)*		1.78 (1.47–2.14)*
> = 36		1.00		1.00		1.00
Sex						
Male		1.03 (0.87–1.22)		1.01 (0.89–1.13)		1.01 (0.90–1.14)
Female		1.00		1.00		1.00
Birth order						
1		1.00		1.00		1.00
2–3		0.91 (0.69–1.19)		0.85 (0.73–1.00)		1.05 (0.87–1.26)
4–5		0.97 (0.71–1.31)		0.82 (0.66–1.01)		1.11 (0.88–1.41)
6+		0.81 (0.54–1.21)		1.01 (0.79–1.29)		1.43 (1.09–1.87)*
Chronic malnutrition						
Yes		0.91 (0.74–1.12)		1.17 (1.02–1.32)*		1.07 (0.93–1.22)
No		1.00		1.00		1.00

*p <0.05 (statistically significant)

The only other maternal variable found to be consistently associated with childhood ARI symptom between 2003 and 2013 was household wealth index. Children from households in the poor wealth quintile were more likely to have ARI symptoms. The outcome was also more likely among children aged less than 36 months. Specifically, the chances of ARI symptoms appeared to greatest in age 12–23 months. Result for 2013 survey also showed that children with birth order six and above were more likely of ARI symptom (OR = 1.43, CI: 1.09–1.22) compared to first births. Under-five children in the North-East, North-West, South-East and South South regions had higher likelihood of ARI symptoms compared to their counterparts in the South-West. These regional differences remained consistent between 2003 and 2013.

## Discussion

Considering the fact that appreciable progress has been made in childhood mortality reduction since 2000 in Nigeria [24], it is expected that similar success must have been recorded for childhood morbidity such as ARI symptoms. This study was aimed at providing empirical evidence in this regard. We described the progress made in reducing the prevalence ARI symptoms among under-five children between 2003 and 2013. Trends in the associated factors were also investigated.

Findings from our study show that the prevalence of ARI consistently reduced from 10.3 to 3.8% in years 2003 and 2013 respectively. This tremendous success may be a result of the substantial progress made globally between the year 2000 and 2015 towards achieving the Millennium Development Goal 4 by member states of the United Nations of which Nigeria is inclusive. The MDGs declaration compels world leaders to reduce child mortality and improve maternal health among other goals [25].

Also, the reduction in the prevalence of ARI symptoms between 2003 and 2013 may be connected to the various intervention efforts of government in ensuring wider immunization coverage for all vaccine-preventable diseases. In the year 2002, Nigeria endorsed the United Nations Special Session (UNGASS) goals of achieving by the year 2010 full immunization of children under 1 year of age at 90% coverage nationally, and with at least 80% coverage at district or equivalent administrative unit levels [26]. Haemophilus Influenza b (Hib) and Pneumococcal conjugate vaccines were introduced into all national immunization programs in years 2006 and 2007 by the WHO [4]. The Nigerian government in 2011 introduced the pentavalent vaccines that protect Hib, pneumonia, and meningitis, in addition to other childhood diseases [4].

Consistent with another study [27], our findings indicated that the proportion of Nigerian households using unclean fuel as their main source of energy increases steadily in the last decade (2003–2013). Solid fuel is the principal domestic fuel globally; a half of world’s population is estimated to use unclean fuels [28]. Nigeria is seen on the global page as poverty-stricken country with a striking gap between the rich and the poor. More than two-thirds of the population live below the recommended poverty line of $1.25 per day [29]. Poverty was named as one of the main drivers for solid fuel use in many developing countries [30].

The use of unclean cooking fuel was not significantly associated ARI symptoms in our study, though it increased the likelihood. Statistical non-significance might be connected to the point that nearly all household use solid fuel, therefore the variation in the sample was not substantial to find significant relationships. Despite the statistical non-significance, young children living in households exposed to unclean fuels have greater risk of developing ARI symptoms compared with those living in households using cleaner fuels or suffering less exposure to smoke and this is consistent with the extant literature [31–33]. The mechanism by which exposure to unclean fuel could induce the occurrence of ARI is partially understood. It’s been noted that exposure to biomass smoke can lead to immune suppression, and increased bronchial reactivity, which enhances one’s vulnerability to bacterial and viral diseases [34, 35].

Though, the probability of ARI symptoms was higher among children exposed to second smoke from adult members who smoke cigarette/tobacco or other substances, also, this did not attain statistical significance. This could be explained by the low numbers of smokers in Nigeria. Previous studies have affirmed that Nigeria has one of the least smoking prevalence globally [36, 37].

Initial models showed that living in houses built with poor quality materials was significantly associated with higher odds of ARI symptoms among under-five children in the year 2008 and 2013. After controlling for other background variables, this variable lost statistical significance, meaning that the adjusted variables were confounders. This does not imply that housing quality is not important for ARI symptoms. Rather, the adjusted variables represent possible pathway in the relationship between housing quality and childhood ARI symptoms. This finding corroborates reports from previous epidemiological studies which showed that living in houses built with inferior materials could result in chronic diseases [38, 39] and childhood mortality [22]. Researchers have established that good housing conditions are indispensable elements for improving household health, and that poor housing and environmental conditions can predispose to many health problems, including infectious diseases (such as tuberculosis and pneumonia) [40, 41].

Our findings are consistent with some previous studies which suggest that ARI symptoms are more likely among young children aged less than 36 months [14, 42]. Specifically, the occurrence of ARI symptoms appearing to greatest in age 12–23 months in years 2003–2013. Compliance with an ample of the period of exclusive breastfeeding and continued breastfeeding in combination with balanced complementary feeding safeguards an infant from ARI. This underscores the significance of the immunoglobulin present in breast milk in protecting against ARI [16].

Also, results from the bivariate and multivariate analysis showed that maternal characteristics associated with higher risk of ARI symptoms in children include poor household wealth quintile. Poverty has previously been linked to increased frequency and severity of ARI symptoms [43]. Also, poverty has consistently been associated with several risk factors such as the use of unclean fuel [44]; sourcing water from unimproved water sources [45]; use of unimproved toilet; crowding, and chronic malnutrition [43]. All these factors have been implicated as risk factors for ARIs symptoms among children.

This study further revealed that under-five children in the South West recorded a lower prevalence of ARI symptoms than those from North East, North West, South East and South South regions. One possible reason for this disparity in the prevalence of ARI symptoms across the geopolitical zones may be the difference in social and economic development in these areas [46, 47]. Earlier studies in Nigeria has affirmed the socio-economic differences between the North and the South [20, 48]. Low immunization coverage, illiteracy, and poverty are more predominant in the North than the South. These factors have a strong influence on disease morbidity and mortality in children [49, 50]. Also, cultural practices like religious beliefs, ethnicity, and politics play a key role in the uptake of routine immunization for vaccine-preventable diseases in the North [51].

We also found that the likelihood of ARI symptoms was higher during the dry season (November to April). The DHS data do not collect variables by which this relationship can be deeply explored. Insights from literature revealed that many of the pathogens for respiratory infections in children are airborne [52]. During dry seasons, these pathogens are often blown around with dust thereby increasing exposures to the risk of infections. The dry season in Northern Nigeria is characterised by a dry and hazy wind due to its geographical location along the course of the Gulf of Guinea [20, 53]. This might further explain why the magnitude of ARI symptoms was higher in Northern Nigeria.

Findings in our study should be interpreted in light of the following limitations. This study was based on self-reported information from mothers of under-five children. The self-report process may introduce some recall bias and subsequently may contribute to an underestimation of ARI symptoms prevalence. In addition, due to the cross-sectional nature of the data, a cause-effect relationship could not be inferred in this study. Another limitation was our inability to include vaccination status among the explanatory variables. Data on vaccination was available only for children less than 36 months whereas we were interested in all children below age five. We did not exclude the older children because they constitute about one-third of the analytical sample. As a result, we were constrained to leave out vaccination status from the analysis. Further, the sample size for 2003 was lower compared to 2008 and 2013 because it was designed to provide only national estimates while the latter two were designed to provide estimates at regional and state levels. Aside difference in sample size, the methodology of data collection and other survey procedures were the same.

The strengths are worthy of mention. First, the uniqueness of this study beyond previous research is its focus on trends in the prevalence and factors associated with ARI symptoms among under-five children in Nigeria between 2003 and 2013. This period coincides with the fastest progress in child survival in Nigeria. The study leverages on the use of the Demographic Health Survey (DHS) data, a nationally representative data whose method of collection has been through a validated process between 1990 and 2013, and its outcome generalizable. Also, variables in the DHS surveys in different countries are defined alike thus allowing for results to be comparable across countries.

## Conclusion

Though this study showed that progress was made in the reduction of the prevalence of ARI symptoms between 2003 and 2013, associated factors have remained the same over the decade. The influence of household environmental factors such as quality of housing material, use of unclean fuel, the presence of a smoker in the household, were partly explained by other variables including age of child and regions of residence. The higher magnitude of ARI symptoms in Northern Nigeria calls for urgent interventions that can guarantee better quality health and survival prospects for under-five children, especially in the most affected regions.

## Abbreviations

ARIs: Acute respiratory infections
CI: Confidence interval
EAs: Enumeration areas
NDHS: Nigeria Demographic and Health Surveys
OR: Odds ratio
UNICEF: United Nations Children’s Fund
WHO: World Health Organisation
